# Open-End Control of Neurite Outgrowth Lengths with Steep Bending Confinement Microchannel Patterns for Miswiring-Free Neuronal Network Formation

**DOI:** 10.3390/mi15111374

**Published:** 2024-11-14

**Authors:** Naoya Takada, Soya Hagiwara, Nanami Abe, Ryohei Yamazaki, Kazuhiro Tsuneishi, Kenji Yasuda

**Affiliations:** 1 Department of Pure and Applied Physics, Graduate School of Advanced Science and Engineering, Waseda University, Tokyo 169-8555, Japan; shikon.purple@fuji.waseda.jp (N.T.); soyahagiwara@ruri.waseda.jp (S.H.); abenanami8011@suou.waseda.jp (N.A.); y.ryohei855@suou.waseda.jp (R.Y.); kazu140613@akane.waseda.jp (K.T.); 2 Department of Physics, School of Advanced Science and Engineering, Waseda University, 3-4-1 Okubo, Shinjuku, Tokyo 169-8555, Japan

**Keywords:** neuron, neurite elongation control, agarose micropattern, bending microchannel array, bending angle, neuronal network formation

## Abstract

Wiring technology to control the length and direction of neurite outgrowth and to connect them is one of the most crucial development issues for forming single-cell-based neuronal networks. However, with current neurite wiring technology, it has been difficult to stop neurite extension at a specific length and connect it to other neurites without causing miswiring due to over-extension. Here, we examined a novel method of wiring neurites without miswiring by controlling the length of neurites in open-ended bending microchannel arrays connected beyond the maximum bending angle of neurite outgrowth. First, we determined the maximum bending angle of neurite elongation to pass through the bending point of a bending microfluidic channel; the maximum angle (the critical angle) was 90°. Next, we confirmed the control of neurite outgrowth length in open-ended microchannels connected at 120°, an angle beyond the maximum bending angle. The neurites stopped when elongated to the bend point, and no further elongation was observed. Finally, we observed that in bending microchannel arrays connected at an angle of 120°, two neurite outgrowths stopped and contacted each other without crossing over the bend point. The results show that the steep bending connection pattern is a robust open-end neurite wiring technique that prevents over-extension and miswiring.

## 1. Introduction

Neuronal network formation is one of the powerful methods that is used to unveil the hidden logical procedures of the brain from the viewpoint of single neurons and the role of their connections. For a long time, many microfabrication techniques have been proposed for patterning geometrically designed neuronal networks. In particular, microprinting and microstructure techniques are two major methods for geometrically controlled neuronal connections [1,2,3,4,5,6,7,8], dendrite-to-axon connections [9,10,11], and for induced neurite differentiation [12]. However, since both techniques are pre-designed with photomasks and stamps, it is difficult to alter the induced neurite connections even after the axonal differentiation of neurites within the designed pattern turns out to be incorrect. In other words, these pre-designed techniques can control the orientation of neurite connections but not the directions.

To overcome the limitations of the above techniques, neuronal wiring technologies have advanced with microfluidic devices, which were pioneered in the 1970s with Campenot chambers [13]. The planar surface gradients of laminin with a three-channel device were applied without microprinting and microstructure techniques for the specific guiding of axons [14]. Several asymmetric microchannel geometries have also been proposed to promote unidirectional axonal outgrowth to develop neuronal networks [15,16]. In addition to the above microfluidic approaches, the structured substrates imposed multiple symmetrical orientations [17]. Indeed, the isotropic structures, including nanowire scaffolds, tend to increase the number of branches by the topography; however, they did not provide direction control for axon elongation and their length control [18,19,20].

We have developed alternative microfabrication techniques to overcome these limitations of conventional microfabrication techniques based on agarose microfabrication technology, which can fabricate microchannels even during cultivation. These techniques melt a portion of a thin agarose layer placed on a culture dish by spot heating with a focused infrared laser and focused micro ion-current without pre-designing [21,22,23]. This flexible agarose microfabrication method allows for additional microfabrication in culture to guide each neurite in the desired direction while observing the neurite outgrowth and differentiation process, which has been difficult with other existing methods.

However, the above constructive method of forming the neural networks on the chip by the stepwise addition of microstructures still presents several difficulties, preventing the realization of a neuronal network for logic calculations with a minimal number of neurons. One of the remaining difficulties is the miswiring of the neuronal network that occurs after the stepwise axon-dendritic connections are formed and during subsequent culture. Other free neurites invade and over-extend through the constructed axon-dendrite connection passages to form unexpected miswiring connections. At present, there are no practical approaches to overcome this miswiring problem using the existing microfabrication technologies.

Therefore, we investigated a method to wire neurites without miswiring by controlling neurite length with an open-end bending microchannel pattern using the maximum bending angle of neurite outgrowth. In this report, we first determined the maximum bending angle of neurite outgrowth to pass through the bending point of the connected two straight microchannels and then examined whether an open-ended steep bending microchannel array can control neurite length at the bending point for neurite connections without causing unexpected additional neurite outgrowth.

## 2. Materials and Methods

This study was conducted in accordance with the Act on Welfare and Management of Animals of the Ministry of the Environment, Japan. All animal experiments and protocols were approved by the Waseda University Animal Experiment Committee (permission numbers: A23-096 and A24-099, respectively) and adhere to ARRIVE 2.0 guidelines.

### 2.1. A 1480-nm Photo-Thermal Etching System

To microfabricate, we used a 1480-nm infrared laser photo-thermal etching system. The system consists of three parts: a phase-contrast microscope (IX-71 with 20× phase-contrast objective lens, LCUPlanFL N, OLYMPUS, Tokyo, Japan), a motorized x-y stage (BIOS-206T, SIGMA KOKI Co., Ltd., Tokyo, Japan), and a 1480-nm focused laser irradiation module (RLM-1-1480, IPG Laser, Oxford, MA, USA). Two wavelengths of light (520 nm visible light for phase-contrast microscopy and 1480 nm laser for photothermal etching) were used simultaneously. Phase-contrast images were acquired using a charge-coupled device CCD camera (CS230, OLYMPUS). The dichroic mirrors and lenses in the system were chosen for their suitability for these two wavelengths.

### 2.2. Agarose Microfabrication

The agarose microstructures on the 35 mm tissue dish (300-035, AGC Technoglass Co., Ltd., Shizuoka, Japan) were prepared as follows. First, the 35 mm dish was made hydrophilic with a plasma ion bombarder (PIB-20, Vacuum Device Inc., Ibaraki, Japan). Then, the bottom of the 35 mm dish was immersed in 100 μL of 1 mg/mL Poly-D-lysine (P0899, Sigma-Aldrich Co., LLC., Tokyo, Japan). After 15 min, the immersed dish was rinsed 3 times with sterilized water to remove liquid Poly-D-lysine and dried for 15 min. Next, 1 mL sterilized water was immersed in the dish, and 650 μL of the water was removed. Then, 85 μL of 3.5% agarose (E-3126-25, BM-BIO BM Equipment Co., Ltd., Tokyo, Japan) was spread on the dish and spin-coated with a spin coater (1H-D7, MIKASA, Tokyo, Japan) at 500 rpm for 3 s and subsequently 3000 rpm for 18 s. After this, 2 mL of water was added to the dish. To lower the temperature of the agarose, the dish was chilled. A 1480 nm laser was spotted on the tip of the water to partially dissolve the agarose layer. Micropatterns were fabricated with the movement of the cultivation dish by operating an automated x-y stage.

### 2.3. Cell Cultivation

Rat hippocampal neurons were isolated and purified from 18-day-old Wister rat embryos (Tokyo Laboratory Animal Science, Tokyo, Japan) using Neuron Dissociation Solutions (291-78001, FUJIFILM Wako Pure Chemical Co., Osaka, Japan). The hippocampal neurons were cultured with neuron culture medium (148-09671, FUJIFILM Wako Pure Chemical Co., Osaka, Japan) on the agarose-patterned Poly-D-lysine-coated 35 mm dish at 37 °C under 5% CO_2_ at saturated humidity. The neurons were placed individually into each round-shaped agarose pattern with a fire-polished glass pipette.

### 2.4. Cell Observation

Neurons on the pattern were observed using inverted optical microscopy (IX-71 with an 20× phase-contrast objective lens, LCPlanFl, OLYMPUS, Tokyo, Japan) with a cooled charge-coupled-device (CCD) camera imaging system (1501M-GE, THORLABS, Newton, NJ, USA).

### 2.5. Immunofluorescence Staining

The immunostaining procedure was conducted using a modified version of a method described in our previous research [22]. For the immunostaining of axons, Anti-Tau-1 (MAB3420, Sigma-Aldrich Co., LLC., Tokyo, Japan) was used as the primary antibody, and Goat anti-Mouse IgG2a Cross-Adsorbed Secondary Antibody, Alexa Fluor 555 (Thermo Fisher Scientific, Waltham, MA, USA) was used as the secondary antibody. For the immunostaining of dendrites, Anti-MAP2 (SIGMA ALDRICH M4403, St. Louis, MO, USA, mouse monoclonal IgG1) was used as the primary antibody, and Rabbit anti-Goat IgG Cross-Adsorbed Secondary Antibody, Alexa Fluor 488 (Thermo Fisher Scientific) was used as the secondary antibody. The fluorescence images were recorded with a cooled charge-coupled device (CCD) camera imaging system (ORCA-ER, Hamamatsu Photonics, Shizuoka, Japan).

## 3. Results and Discussion

### 3.1. Fabrication of Angled Single Neurite Elongation Patterns

**Figure 1:**
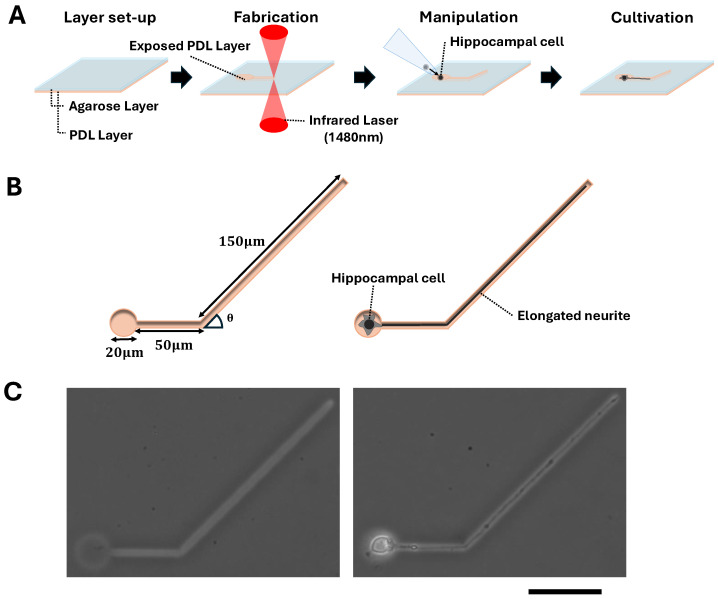
Fabrication of angled single neurite elongation patterns in agarose microstructures. (**A**) Schematic drawing of angled agarose microchannels fabrication procedure in a thin agarose layer on the cultivation dish. First, a thin agarose layer is coated on the poly-D-lysine colated cultivation dish. Then, the focused infrared laser is applied to the agarose layer to melt a portion of the agarose for the microchamber and angled microchannel formation. A hippocampal cell is set in the microchamber. Finally, the elongation of neurite in the angled microchannel was observed. (**B**) Schematic images of the microchamber and angled microchannel for single neurite elongation observation. Left, design of the microchamber and angled microchannel pattern. Right, a neuron is placed in the microchamber, and the elongated neurite is elongated in the angled microchannel. (**C**) Micrographs of the microchamber and the 45° angled bending microchannel before (**left**) and after the neurite elongation cultivation started (day 3) (**right**). Bar, 50 μm.

We first evaluated the ability of neurite outgrowths to pass through the bending microchannels. For that, we fabricated agarose micropatterns with 3 μm wide narrow bending microchannels with various bending angles in the thin agarose layer coated on the cultivation dish, exploiting the 1480 nm photo-thermal etching system. (Figure 1A). After the bending microchannels were fabricated, single neurons (hippocampal cells) were placed in the 20 μm microchambers connected to the bending microchannels at their ends. The extension of the neurites in the bending microchannels was observed, and the maximum bending angle at which the neurites can extend from the bending point was examined.

Figure 1B,C show a schematic and a micrograph of a microchannel bent at a 45° angle and neurite outgrowth in it. In this example, the bending microchannel was designed to extend 50 μm straight from the microchamber, then be bent 45° and connect to the second 150 μm straight microchannel. The first neurite was elongated within this 45° bending microchannel and extended beyond the bending point to the end of the 150 μm second straight microchannel. Similarly, using these bending microchannel pathways, neurite outgrowth passing through the bending point was observed at various bending angles from 20° to 120° degrees.

### 3.2. Measurement of Angle Degree Dependence of Single Neurite Elongation

**Figure 2:**
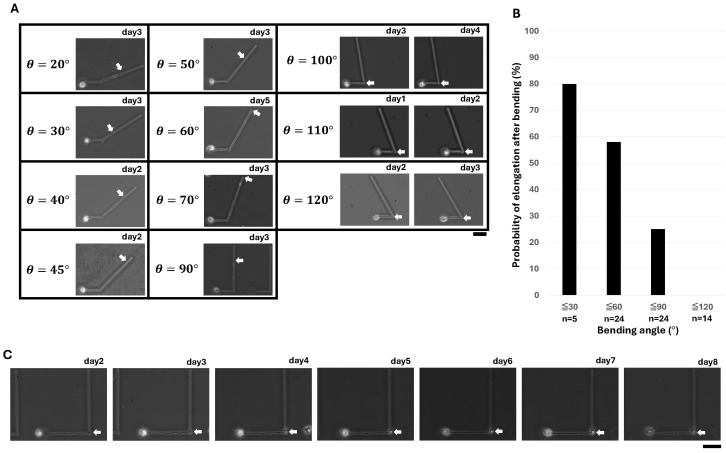
Ability of single neurite elongations in various bending angles. (**A**) Neurite elongations in the various bending microchannels from 20° to 120° bending angles. Neurites in the bending angles larger than 90° stopped their elongation at the bending point. To confirm that they had stopped, two micrographs were compared: left, the day elongation stopped; right, a day after to confirm their stopping. (**B**) Summary of bending angle dependence of neurite elongations. (**C**) Observation of the neurite outgrowth until six days (day 8) after its elongation stopped at the 90° bending point (day 2). White arrows indicate the position of the neurite tips. Bars, 50 μm.

Figure 2A shows the micrographs of neurite outgrowth at the bending angles from 20° to 120°. Under normal cultivation circumstances, the neurites would elongate and pass the bending point by 2 to 3 days after the cultivation started, so if they had passed the bending point by that time, the neurites were judged to have passed the bending point at this angle. Otherwise, if the tip of the neurite seemed to stop at the bending point, an elongation of the tip of the neurite was examined the next day. If no extension was observed, the neurite was considered to have failed to pass through this angle. As shown in the micrographs, we observed the samples passing through the bending point from 20° to 90° angles. However, at angles greater than 90°, no neurites passed through the bending point.

Table 1 summarizes the total experimental results of neurite outgrowth at various bending angles. Up to a bending angle of 90°, neurite outgrowths were observed passing through the bending point. However, when the bending angle was greater than 90°, outgrowth was hindered and stopped at the bending point. The results are also replotted in Figure 2B. As shown in the graph, as the bending angle increased, the percentage of elongation passing through the bending point gradually decreased, and finally, when the bending angle was greater than 90°, the neurites no longer elongated past the bending point.

Figure 2C shows an example of the duration of the stopped neurite at the critical angle, the 90° bending point. As shown in Table 1, 74% of the neurites, including this sample, stopped at the 90-degree bending points. As shown in the micrographs, after the neurite reached the bending point and stopped (day 2), the tip of the neurite remained, even six days after its stopping.

These results indicate two critical rules in neurite network design: One is that the probability of the neurite elongation passing through the bending point is reduced even when the bending angle is less than 90°; another is that when the bending angle of the microchannel is more than 90°, the sudden bending stops the elongation of the neurite outgrowth. The former rule is extremely important in designing the wiring of neurites; this rule says we should design neurite networks without bending corners in the microchannels. The latter rule is most important for the development of miswiring-free neurite wiring technology; this rule suggests that a bending path with a large bending angle of more than 90° can stop neurite outgrowth at the bending point and prevent miswiring in open-end microchannels for neurite network patterning. In this sense, the angle of 90° can be regarded as the critical angle of outgrowth in the bending patterns.

### 3.3. Effect of Bending Angles for Neurite Differentiation

Using the angled bending microchannel patterns, we found two rules of neurite outgrowth at the bending point as a part of the fundamental neurite network designing rules. Rule one is that the elongation rate decreases with the increase in bending angle, even though the neurites can elongate through the bending point of up to 90° bending angles. Hence, as described above, rule one recommends using curved microchannels rather than bending patterns to change the direction of neurite elongations. Then, what else affects the neurites by these bending patterns, especially in their functions? We examined the effect of the bending pattern on the axonal differentiation of neurites.

**Figure 3:**
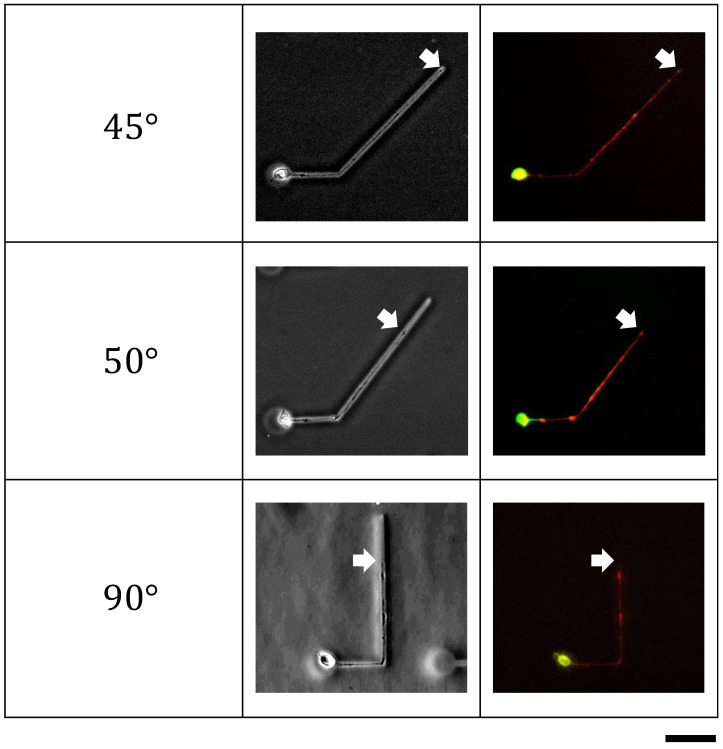
Effect of bending angles for single neurite differentiation. (**Left**), bending angles. (**Middle**), phase-contrast images of neurons and their neurites in the microchambers and the bending microchannels. (**Right**), fluorescent images of immunostained neurons and neurites: neurons and neurites were fixed on day 2 (45°) or day 3 (50° and 90°) after the cultivation started, and were immunostained for Tau-1 to indicate axons (red) and for MAP-2 to indicate cell bodies and dendrites (green). The white arrows show the positions of the elongated neurite tips. Bar, 50 μm.

Figure 3 shows the results of the effect of the bending patterns for the axonal differentiation of neurites. After two (45°) or three days (50° and 90°) of cultivation in the bending microchannels, all the first neurites elongated from the neurons passed through the bending point and differentiated into axons. Thus, we can conclude that at least the bending pattern does not affect the axonal differentiation of the extending neurites.

The ability of axonal differentiation in the bending structures implies substantial evidence of transportation mechanisms in bending neurites. The axonal differentiation requires microtubule-assisted kinesin/dynein transportation [24,25,26]. Therefore, this evidence may support that the steep bending does not affect the transportation of other proteins in the neurites, at least in the transportation of proteins similar to those for axonal differentiation [27]. However, the mechanism of axonal differentiation itself, especially the dominant factors that determine the fate of axonal differentiation, is still under exploration. Therefore, the effects of bending patterns on axonal differentiation should also be examined in the future [28].

### 3.4. Constructive Arrangement of Neurite Outgrowth Length Control Using Angled Bending Shapes of an Open-End Microtunnel Structure

Rule two of neurite outgrowth in the bending patterns indicated that the steep bending patterns can control the outgrowth length of neurites, even in the open-end microchannels. To confirm this ability, we next examined the outgrowth length regulation with the steep bending patterns of microchannels.

**Figure 4:**
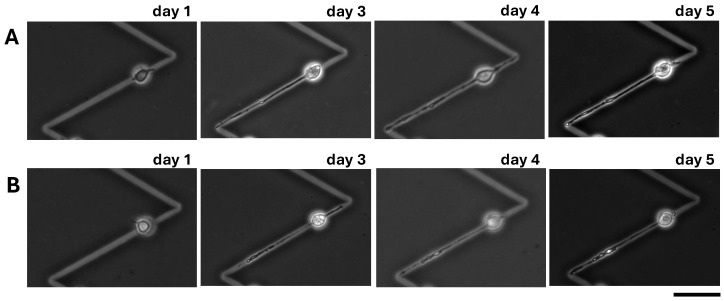
Neurite outgrowth length control using angled bending shapes of open-end microtunnel structure. (**A**,**B**) Phase-contrast images of neurites elongated from single neurons in the 120° steep bending open-end microchannel series. Microchamber diameter, 20 μm; microchannel lengths, 110 μm (left side), and 30 μm (right side). Bar, 50 μm.

Figure 4 shows the neurite outgrowth control experiment results. The microchamber for placing a neuron was set on the straight microchannel, and both ends of the microchannel were connected to other straight microchannels with steep 120° bending angles. In both micrographs, the first neurites elongated from neurons and almost reached the bending point on day 3, and they stopped at the bending points even two days later (day 5). The second neurites also started elongation, and both of the second neurites reached another bending point on day 3 or day 4. The first neurite elongated to the end of the straight microchannel to the bending point and maintained its elongated shape during the entire observation periods until day 5. However, the second neurites were unstable in maintaining their elongated form and shrunk after reaching the bending point.

The above results indicate that the steep bending angle can control the outgrowth length of neurites at the bending points, even though the microchannels are open-ended for connecting other neurites elongated from other neurons when the bending angles are larger than the critical angle, 90°.

### 3.5. Application of 120° Steep Bending Microchannel Series for Connecting the Length-Controlled Neurites

As the neurite outgrowth length control using the 120° steep bending patterns was accomplished, we examined the connection of neurites at the bending point of the 120° steep bending pattern series.

**Figure 5:**
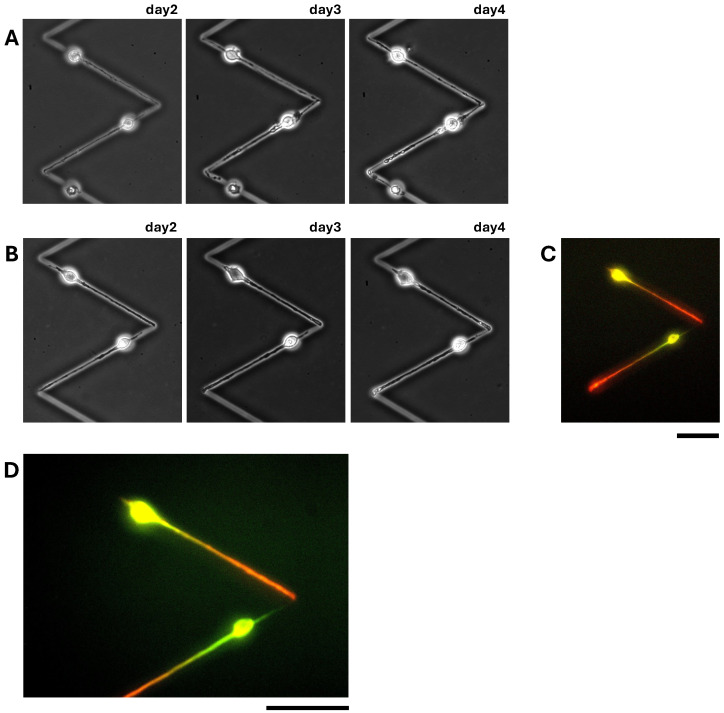
Connection of length-controlled neurites using 120° steep bending microchannel series. (**A**,**B**) Phase-contrast images of neurites elongated from neurons in the 120° steep bending open-end microchannel series. (**C**) A fluorescent image of immunostained neurons and neurites of (**B**) neurons and neurites were fixed on day 4 and immunostained for Tau-1 to indicate axons (red) and for MAP-2 to indicate cell bodies and dendrites (green). (**D**) A magnified fluorescent image of immunostained neurons and neurites of (**C**) with 40× obj. lens. Bars, 50 μm.

Figure 5 shows the results of the neurite connection experiment. As shown in the micrographs in Figure 5A,B, the first neurites elongated in the straight microchannels to their bending points and stopped (day 2). Then, the following second neurites elongated from neurons were also elongated to the bending points and contacted (day 3), and their connections were maintained (day 4). To confirm the function of bending angles for preventing over-extension even after the connection of two neurites, we checked their elongations with the immunostaining of neurites (Figure 5C,D). The fluorescence image showed that the two first neurites were stained in red, indicating axon, and remained in the bending point of the bending patterns even two days after their connections were formed. Therefore, we concluded that the 120° steep bending microchannel series worked sufficiently to connect neurites, maintaining their elongation border without over-extension at the open-end connections.

As shown in this paper, we have developed a method of neurite wiring without miswiring by controlling the neurite length in open-ended microchannels connected beyond the maximum bending angle of neurite extension. The validity of the critical angle of 90° was also confirmed. For the next step, we need to continue further to extend the design principles of neuronal network formation in addition to our proposed two rules of outgrowth in flexion patterns, etc., in order to accomplish single-cell-based neuronal network operations.

## 4. Conclusions

We investigated a novel method of wiring neurites without miswiring by controlling the length of neurites within open-ended linear microchannels connected at an angle larger than the maximum bending angle of neurite extension. First, we clarified the maximum bending angle of neurite extension to pass through the bending point of a bent microchannel. The bending angle dependence of the neurite elongation was examined from 20° to 120°, and the maximum angle (critical angle) of neurite elongation to pass through was 90°, at which 26% of neurites passed the bending point. The effect of bending on axonal differentiation was also examined using immunostaining, showing that even after experiencing 90° bending, the entire elongated neurites differentiated into axons. Then, we confirmed the control of neurite outgrowth length in open-ended microchannels connected at an angle beyond the maximum bending angle, 120°. All neurites elongated and stopped at the bend points, and no further over-extension was observed. Finally, we observed that in two microchannels connected at an angle of 120°, two elongated neurites stopped and contacted each other at the bending point without crossing over the bend point. The results indicate that the steep bending connection can control neurite outgrowth lengths and, therefore, can work as the new method of neurite wiring without miswiring due to over-extension.

## Figures and Tables

**Table 1:** Single neurite elongation in various bending angles of microchannels.

Bending Angle (°)	Elongated	Stopped	Probability of Elongation After Bending (%)	Number of Samples
20	1	0	100	1
30	3	1	75	4
40	3	2	60	5
45	4	0	100	4
50	6	2	75	8
60	1	6	14	7
70	1	4	20	5
90	5	14	26	19
100	0	5	0	5
110	0	2	0	2
120	0	7	0	7

## Data Availability

The original contributions presented in the study are included in the article, further inquiries can be directed to the corresponding author.
